# An Investigation into the Influence of Graphene Content on Achieving a High‐Performance TiO_2_‐Graphene Nanocomposite Supercapacitor

**DOI:** 10.1002/open.202400128

**Published:** 2024-07-31

**Authors:** Negar Naghavi, Maisam Jalaly, Samira Mohammadi, S. Morteza Mousavi‐Khoshdel

**Affiliations:** ^1^ Nanotechnology Department School of Advanced Technologies Iran University of Science & Technology (IUST), Narmak Tehran 16846-13114 Iran; ^2^ School of Chemistry Iran University of Science & Technology (IUST), Narmak Tehran 16846-13114 Iran

**Keywords:** TiO_2_ nanoparticles, Graphene, Hydrothermal, Supercapacitor

## Abstract

This study presents the synthesis of TiO_2_‐graphene nanocomposites with varying mass ratios of graphene (2.5, 5, 10, 20 wt. %) using a facile and cost‐effective hydrothermal approach. By integrating TiO_2_ nanoparticles with graphene, a nanomaterial characterized by a two‐dimensional structure, unique electrical conductivity and high specific surface area, the resulting hybrid material shows promise for application in supercapacitors. The nanocomposite specimens were characterized by X‐ray diffraction (XRD), Fourier transform infrared (FTIR) spectroscopy, Raman microscopy, field‐emission scanning electron microscopy (FESEM), and transmission electron microscopy (TEM). Additionally, supercapacitive properties were investigated using a three‐electrode setup by cyclic voltammetry (CV), galvanostatic charge‐discharge (GCD) and electrochemical impedance spectroscopy (EIS) tests. Notably, the TiO_2_‐20 wt. % rGO nanocomposite exhibited the highest specific capacitance of 624 F/g at 2 A/g, showcasing superior electrochemical performance. This specimen indicated a high rate capability and cyclic stability (93 % retention after 2000 cycles). Its remarkable energy density and power density of this sample designate it as a strong contender for practical supercapacitor applications.

## Graphical abstract

TiO_2_‐rGO nanocomposite was in‐situ synthesized by one pot hydrothermal method. Electrochemical studies of the electrode material was conducted to assess its potential for supercapacitance applications. The effect of graphene content in the nanocomposite on the supercapacitive performance was investigated. TiO_2_‐20 wt. % rGO nanocomposite exhibited an exceptional specific capacitance and energy density.

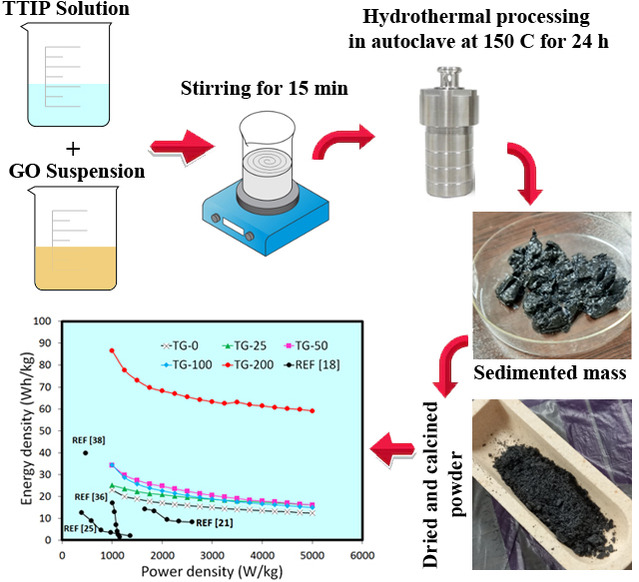

## Introduction

With increasing power and energy demands across various sectors in recent decades, the future world is poised to encounter an inevitable energy shortage crisis. In addition, the emissions generated by the combustion of fossil fuels pose a significant threat to the environment, exacerbating pollution levels. This looming crisis necessitates a shift towards renewable energy sources like solar, wind, and marine energy for sustainable solutions. Simultaneously, there is a crucial need for efficient energy storage systems to ensure an optimal balance of power sourced from these channels.

Supercapacitors represent a novel energy storage technology that has attracted great attention due to their remarkable attributes, including high power density, rapid charge/discharge rates, excellent reversibility, and cyclic stability.[^1^, ^2^] These devices can be classified into three distinct categories based on their charge storage mechanisms. The first category consists of electric double‐layer capacitors (EDLC), typically fabricated using carbon‐based materials such as activated carbon, carbon nanotubes, and graphene for their high surface area and long cyclic stability.[^3^, ^4^] The second category is pseudocapacitor supercapacitors composed of transition metal oxide and conductive polymers.[^5^, ^6^] RuO_2_, MnO_2_, NiO, V_2_O_5_, Co_3_O_4_, and TiO_2_, are known for their high specific capacitance, energy density, and power density, which are widely explored in research on pseudocapacitor supercapacitors.[^7^, ^8^, ^9^] The third type encompasses hybrid supercapacitors, combining the advantages of both EDLCs and psuedocapacitors. This hybrid charge storage mechanism involves a combination of Faradaic and non‐Faradaic reactions, typically utilizing nanocomposites for the supercapacitor cells. Combining the advantages of the preceding types, hybrid supercapacitors have demonstrated enhanced electrochemical performance in terms of specific capacitance, energy density, power density and cyclic stability. These properties make hybrid supercapacitors so useful for diverse energy applications.[^1^, ^10^, ^11^]

Titanium dioxide (TiO_2_) stands out as a highly promising material for the supercapacitor electrodes due to its stable electrochemical properties, affordability, non‐toxicity and ease of production. Furthermore, the multiple oxidation states of TiO2, as a transition metal, allow it to participate in reversible reduction and oxidation reactions during the charge and discharge cycles of energy storage devices. This redox activity can enhance the energy storage capacity and cycling performance of these materials when used in applications like batteries, supercapacitors, and electrochemical energy storage systems. However, its specific capacitance decreases significantly with increasing scan rate. On the other hand, graphene, composed of a single layer of densely packed sp^2^‐hybridized carbon atoms in a hexagonal lattice structure, emerges as a viable electrode material for supercapacitors. This is due to its remarkable electrical characteristics, ultra‐high specific surface area, and robust chemical stability.[^9^, ^12^, ^13^]

A limited number of studies have thus far explored TiO_2_‐graphene nanocomposites for supercapacitor applications. Ramadoss et al.^[14]^ synthesized a hybrid structure comprising reduced graphene oxide /TiO_2_ nanorods/reduced graphene oxide (rGO/TiO_2_ NR/rGO) by spin coating and hydrothermal method. This nanocomposite exhibited a specific capacitance of 114.5 F/g at a scan rate of 5 mV/s and maintained 85 % capacitance retention after 4000 cycles. The study conducted by Yoruk et al.^[15]^ showed that rGO/TiO_2_/PANI nanocomposite has a significantly better supercapacitor performance compared to each individual components. The enhanced performance was reflected in its improved specific capacitance, energy density, power density, and cyclic life. Furthermore, an rGO‐TiO_2_ nanocomposite with a constant mass ratio of 7 : 3 was synthesized using a combination of modified Hummers and hydrothermal techniques.^[16]^ Their results revealed that rGO‐TiO_2_ nanobelts and rGO‐TiO_2_ nanoparticles had specific capacitances of 225 and 60.8 F/g at a discharge current density of 0.125 A/g, respectively. The higher capacitance of nanobelt structure was attributed to its unique morphology, enhanced charge transfer capabilities, and increased contact area with rGO nanosheets. Sun et al.^[17]^ utilized the atomic layer deposition (ALD) method to deposit TiO_2_ nanocoating onto graphene nanosheets, resulting in specific capacitances of 75 and 84 F/g at a scan rate of 10 mV/s for composites grown through 50 and 100 ALD cycles, respectively. Additionally, when compared to TiO_2_ nanoparticles (NPs), one‐dimensional TiO_2_ nanowires (TiO_2_ NWs) have higher specific capacitance, larger specific surface areas, and enhanced contact areas with the electrolyte solution. Remarkably, TiO_2_ NWs can be woven into a network configuration, providing channels for the directional ion transfer.[^18^, ^19^]

These studies collectively underscore the significant potential of TiO_2_‐graphene nanocomposites for supercapacitor applications. Therefore, in the present research, TiO_2_‐graphene nanocomposites with varied mass ratios of graphene were synthesized via the hydrothermal method. The study were meticulously examined the influence of graphene weight fraction in the nanocomposite composition on its structural characteristics and electrochemical performance.

## Results and Discussion

**Figure 1:**
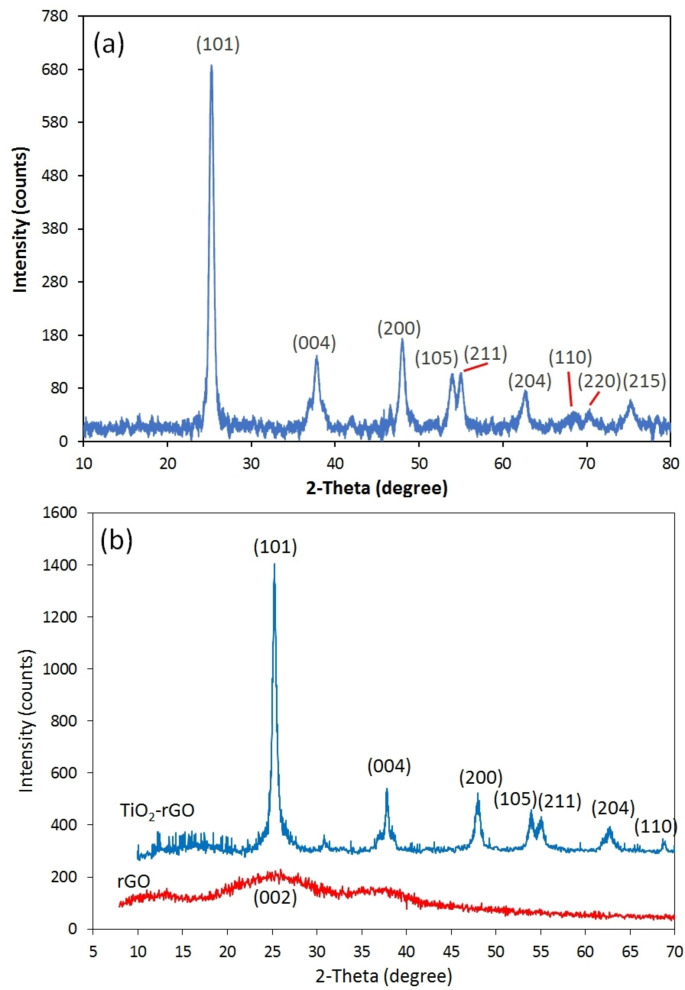
XRD patterns of (a) TG‐0, (b) rGO and TG‐200.

The XRD patterns were utilized to investigate the crystallinity, purity, and particle size of the synthesized products. Figure 1 shows the XRD patterns of as‐prepared TiO_2_ and TiO_2_‐rGO nanocomposite. Well‐defined crystalline peaks located at 25.3°, 37.7°, 48.2°, 56.6° and 75.1° correspond to the (101), (004), (200), (211) and (215) crystalline planes of anatase polymorph of TiO_2_ (JCPDS Card no: 01‐071‐1167). Significantly, no other phases were detected in Figure 1(a). This figure clearly illustrates that at the calcination temperature of 550 °C, the singular phase present for TiO_2_ nanoparticles is anatase, which has been recognized as the superior phase for titania with the best electrochemical properties.[^20^, ^21^]

The XRD pattern of the TiO_2_‐rGO nanocomposite (TG‐200 sample) is shown in Figure 1(b). In addition, pure graphene oxide (GO) was subjected to the identical hydrothermal processing and heat treatment to generate reduced graphene oxide (rGO) for comparative analyses (see Figure 1(b)). As can be observed, the anatase phase was detected within the nanocomposite sample as well. In the XRD diffraction pattern of rGO, the primary peak observed at ~26° is attributed to the (002) plane of rGO, which matches well with diffraction spectra of graphite (JCPDS Card no: 00‐041‐1487). This peak is not detected in the XRD pattern of the nanocomposite sample, apparently due to its overlap or suppression by the sharp (101) peak of anatase TiO_2_, which is consistent with previous studies.[^21^, ^22^, ^23^] Using the Scherrer equation, the crystallite size of the TiO_2_ nanoparticles was calculated to be approximately 56 nm.

**Figure 2:**
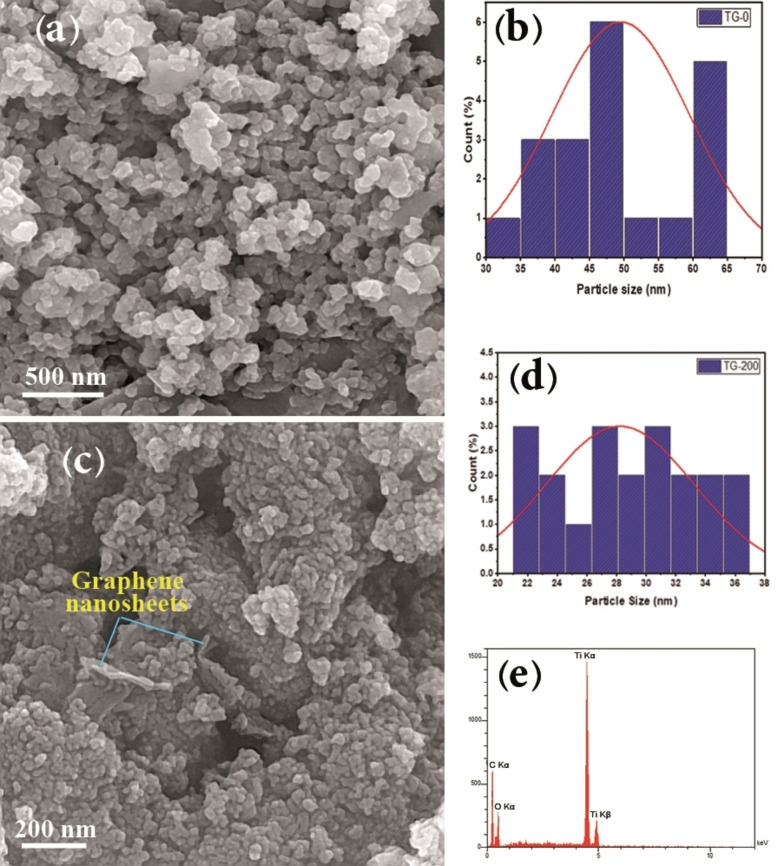
FESEM image (a) and particle size distribution (b) of TG‐0 sample. FESEM image (c), particle size distribution (d) and EDX analysis of TG‐200 sample.

The FE‐SEM micrographs of TG‐0 and TG‐200 samples are presented in Figure 2. The size and surface morphology of nanoparticles and nanocomposites can significantly impact the performance of electrodes utilized in supercapacitors. This provides valuable insights into the active sites required for ion migration between the electrode and electrolyte. A larger surface area of electrode materials is effective in offering a greater number of electroactive sites, even at high current density.^[24]^ Moreover, the size of the active materials used in supercapacitor electrodes plays a key role in determining the specific capacitance. Nanostructures with increased surface area can minimize the transfer paths of electrons and ions, thereby facilitating ion diffusion.^[25]^ Hence, the production of nanoparticles with reduced diameters is crucial.

Notably, semi‐spherical titanium dioxide nanoparticles are clearly detectable in Figure 2(a). The particle size distribution of TiO_2_ nanoparticles is illustrated in Figure 2(b), revealing an average diameter of about 49 nm. Figures 2(c) and (d) show the FE‐SEM micrograph and particle size distribution of the TG‐200 sample (TiO_2_‐20 wt. % rGO). Two‐dimensional graphene nanosheets and TiO_2_ spherical nanoparticles are well distinguished. TiO_2_ nanoparticles, with an average diameter of 28 nm, are uniformly distributed within the graphene nanosheets. In addition, area EDX analysis in Figure 2(e) verifies the presence of Ti, O, and C elements, confirming the combination of TiO_2_ and graphene phases in the composite TG‐200 sample.

**Figure 3:**
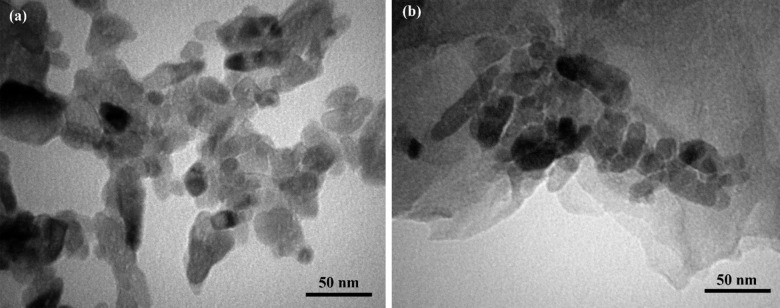
TEM images of (a) TG‐0 and (b) TG‐200.

Transmission electron microscopy (TEM) is the preferred technique to acquire precise measurements of nanoparticle diameters, grain size, particle distributions, and morphologies.^[5]^ The TEM micrographs of the prepared TG‐0 and TG‐200 samples are depicted in Figure 3. The TiO_2_ nanoparticles exhibit a semi‐spherical morphology. Two‐dimensional graphene nanosheets decorated with TiO_2_ nanoparticles are also observed in Figure 3(b).

**Figure 4:**
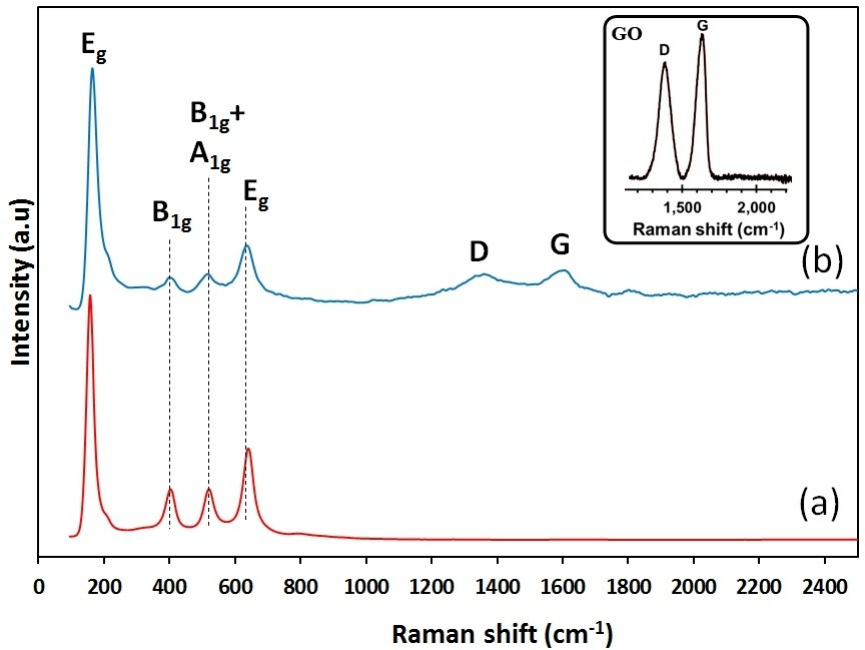
Raman spectroscopy of (a) TG‐0 and (b) TG‐200. The inset shows the Raman spectrum for graphene oxide.

To further confirm the crystalline quality and the formation of the chemical bonds in the TiO_2_ nanoparticles and their combination with graphene, Raman analysis was employed. Raman spectra of pure TiO_2_ and TiO_2_ nanoparticles loaded with 20 wt. % graphene are shown in Figure 4. Four specific Raman bands are located at *c.a*. 150, 400, 515, and 640 cm^−1^, which correspond to the E_g_, B_1g_, A_1g_+B_1g_, and E_g_ vibration modes of the anatase phase of TiO_2_.[^26^, ^27^, ^28^] Particularly noteworthy is the sharply defined and intense Raman band at 150 cm^−1^, solidifying the successful synthesis of TiO_2_ in its anatase form.

**Figure 5:**
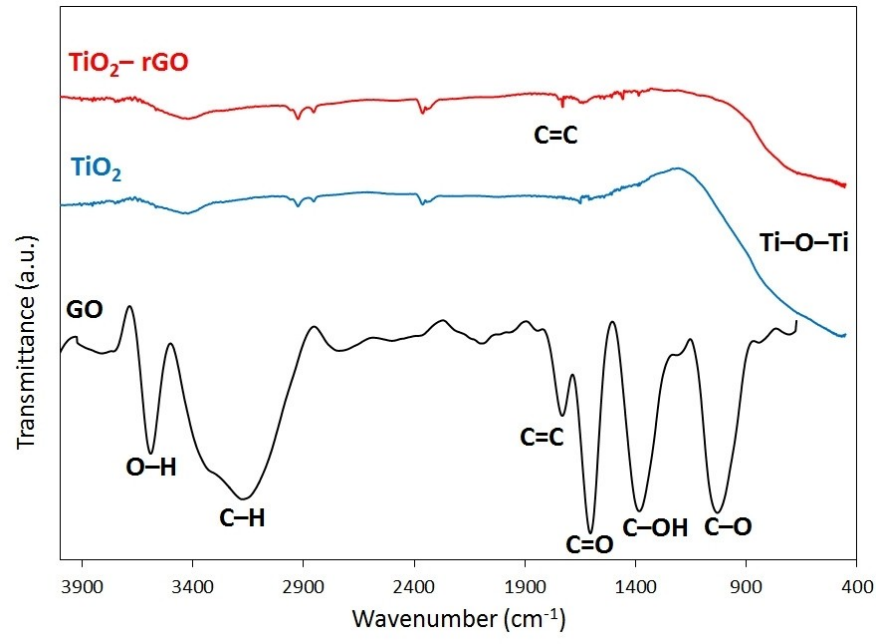
FTIR spectra of GO, TG‐0 and TG‐200 samples.

Furthermore, the Raman spectrum of the TiO_2_ nanocomposite containing 20 wt. % rGO (TG‐200) is presented in Figure 4. In addition to the characteristic peaks of TiO_2_, two distinct peaks are observable, i. e. the disorder peak (D peak at *c.a*. 1359 cm^−1^) and the graphitic peak (G peak at *c.a*. 1560 cm^−1^). The D band is attributed to the presence of defects and lattice disorder/deformation, while the G peak signifies the sp^2^‐bonded carbon atoms within the 2‐D hexagonal graphitic layer of the graphene structure.[^21^, ^29^] The intensity ratio of the D and G bands (I_D_/I_G_) serves as a criterion for the evaluation of the graphitization degree of the graphene surface, as well as the disorder and defects within the carbon network.^[30]^ The inset in Figure 5 displays the D and G bands of the provided graphene oxide (GO). The I_D_/I_G_ parameter increased from 0.85 for GO to 1.02 for rGO, suggesting a decrease in oxygenated functional groups on the GO nanosheets following the reduction processing and an increase of the density of residual defects resulting from removal of functional moieties.[^30^, ^31^] This observation confirms the conversion of graphene oxide into reduced graphene oxide at 550 °C.

Figure 5 shows the FTIR spectra of GO, TG‐0, and TG‐200 samples. The transmittance peaks of the GO spectrum, appeared at wavenumbers of about 1730, 1600, 1380, and 1058 cm^−1^, are related to the stretching vibrations of the C=C bond, O=C carbonyl group, C−OH group, and C−O bond within the epoxy group, respectively.[^32^, ^33^] In addition, the prominent bands observed at around 3100 and 3600 cm^−1^ are ascribed to the stretching vibration of C−H and O−H bonds, respectively. In case of the TiO_2_ sample, a significant peak assigned to the stretching vibration of the Ti−O−Ti bond is detected at about 470 cm^−1^. Additionally, the peak observed near 3400 cm^−1^ is attributable to the hydroxyl group originating from adsorbed H_2_O molecules. The characteristic bands of GO exhibited a substantial decrease or disappearance in the nanocomposite sample (TiO_2_‐rGO) following the reduction of graphene oxide. A minor C=C skeletal vibration at about 1730 cm^−1^ is also evident, confirming the presence of graphene within the nanocomposite sample.

**Figure 6:**
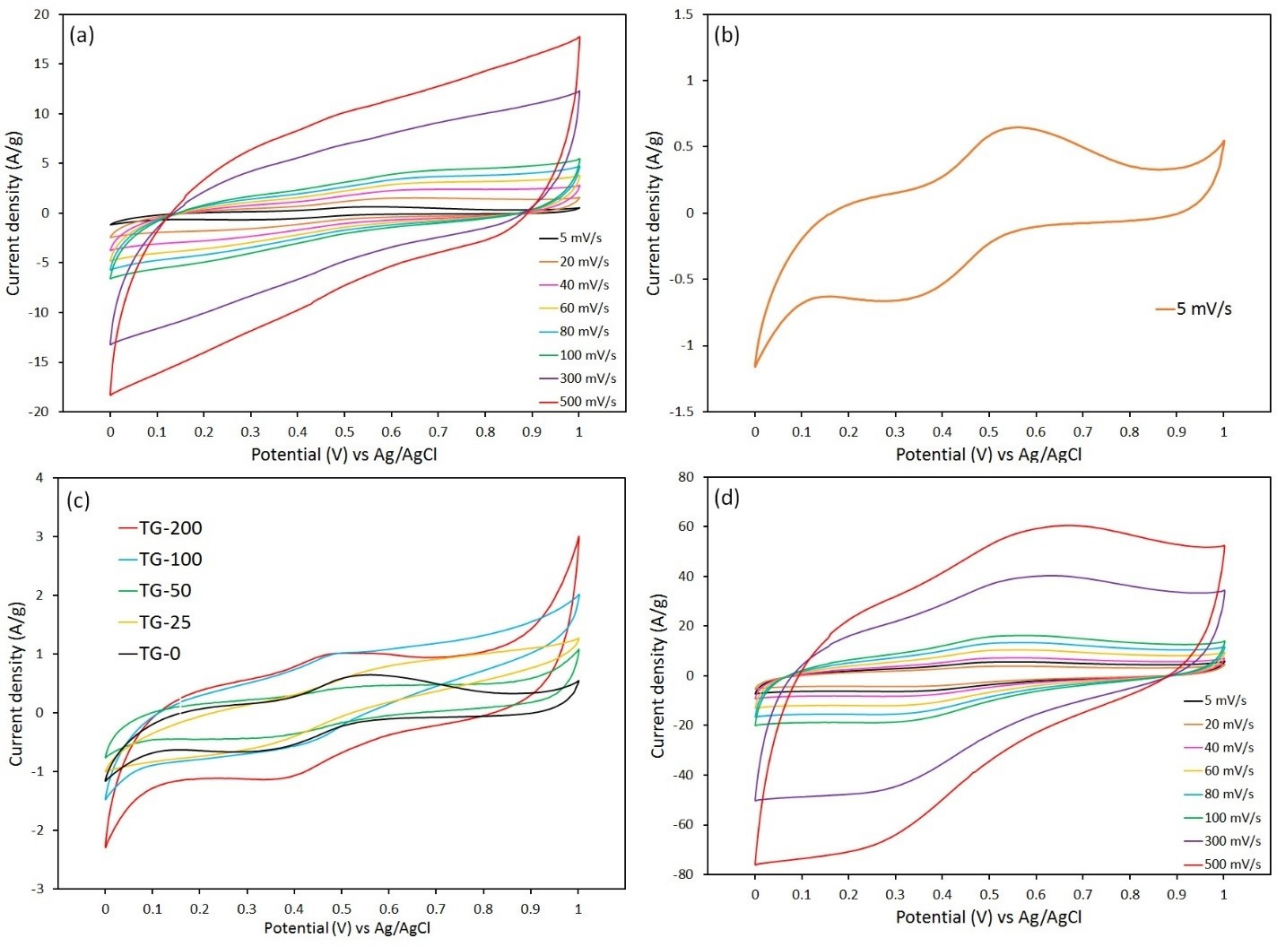
CV curves of (a) TG‐0 sample at different scan rates, (b) TG‐0 sample at 5 mV/s, (c) all nanocomposite samples at 5 mV/s, (d) TG‐200 sample at different scan rates.

CV and GCD measurements were accomplished to evaluate the specific capacitance, energy density, and power density of the fabricated supercapacitors. The CV curves of the TiO_2_ nanoparticles were obtained at different scan rates within the potential window of 0–1 V, as shown in Figure 6(a). It is evident that the CV curves of the sample exhibited symmetrical, rectangular‐like shapes with very small redox peaks, indicating the electrical double‐layer capacitance behavior and rapid charging/discharging process (Figure 6(b)). This suggests the coexistence of redox and capacitive reactions within the supercapacitor. Moreover, the internal area of the CV curves significantly increases with increasing scan rate, confirming the non‐Faradaic behavior of the supercapacitor and its excellent rate capability.[^21^, ^34^]

Furthermore, the CV curve of TG‐0 sample at 5 mV/s is depicted in Figure 6(b). In this curve, the oxidation and reduction peaks are clearly visible. Notably, these peaks demonstrate a nearly symmetrical pattern, underscoring the high reversibility of the electrode composed of TiO_2_ nanoparticles. The presence of these oxidation and reduction peaks signifies the occurrence of redox reactions at the electrode‐electrolyte interface, indicative of the pseudocapacitive behavior of the electrode material. This behavior of TiO_2_ originates from a Faradaic charge storage mechanism, which initiates reversible redox reactions on the electrode surface through the transport of ions within the electrolyte solution. Redox reaction in TG‐0 electrode occurs according to the following reaction:^[35]^


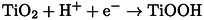




The electrochemical performance of nanocomposite samples with different graphene content at different scan rates was also investigated. Figure 6(c) shows the CV curves obtained from TG‐0, TG‐25, TG‐50, TG‐100 and TG‐200 samples at a scan rate of 5 mV/s. As can be observed, the nanocomposites exhibited a larger rectangular CV curves of in comparison with TG‐0 sample. In fact, the increased area of the rectangular CV curves in the nanocomposites is manly attributed to the synergistic interplay of Faradaic contribution (psuedocapacitance effect arising from TiO_2_) and non‐Faradaic contribution (EDLC effect arising from graphene). Moreover, it can be seen that increasing the graphene content in the nanocomposite samples resulted in an enlargement of CV window. Among all samples, the TG‐200 sample (TiO_2_‐20 wt. % rGO) exhibited the largest internal area within the CV curve, implying superior electrochemical performance. Thus, the CV analysis of the TG‐200 sample was conducted at different scan rates, as shown in Figure 6(d). Notably, all curves maintained the semi‐rectangular shape even at high scan rates, suggesting the characteristic of ideal capacity and excellent reversibility of the electrode. As the scan rate increases, the oxidation and reduction peaks shift towards higher and lower potentials, respectively. This phenomenon leads to the improvement of polarization effects and kinetics associated with the transfer of electrolyte ions to the electrode interfaces, thereby corroborating the enhanced electrochemical performance of the nanocomposites.^[25]^

**Figure 7:**
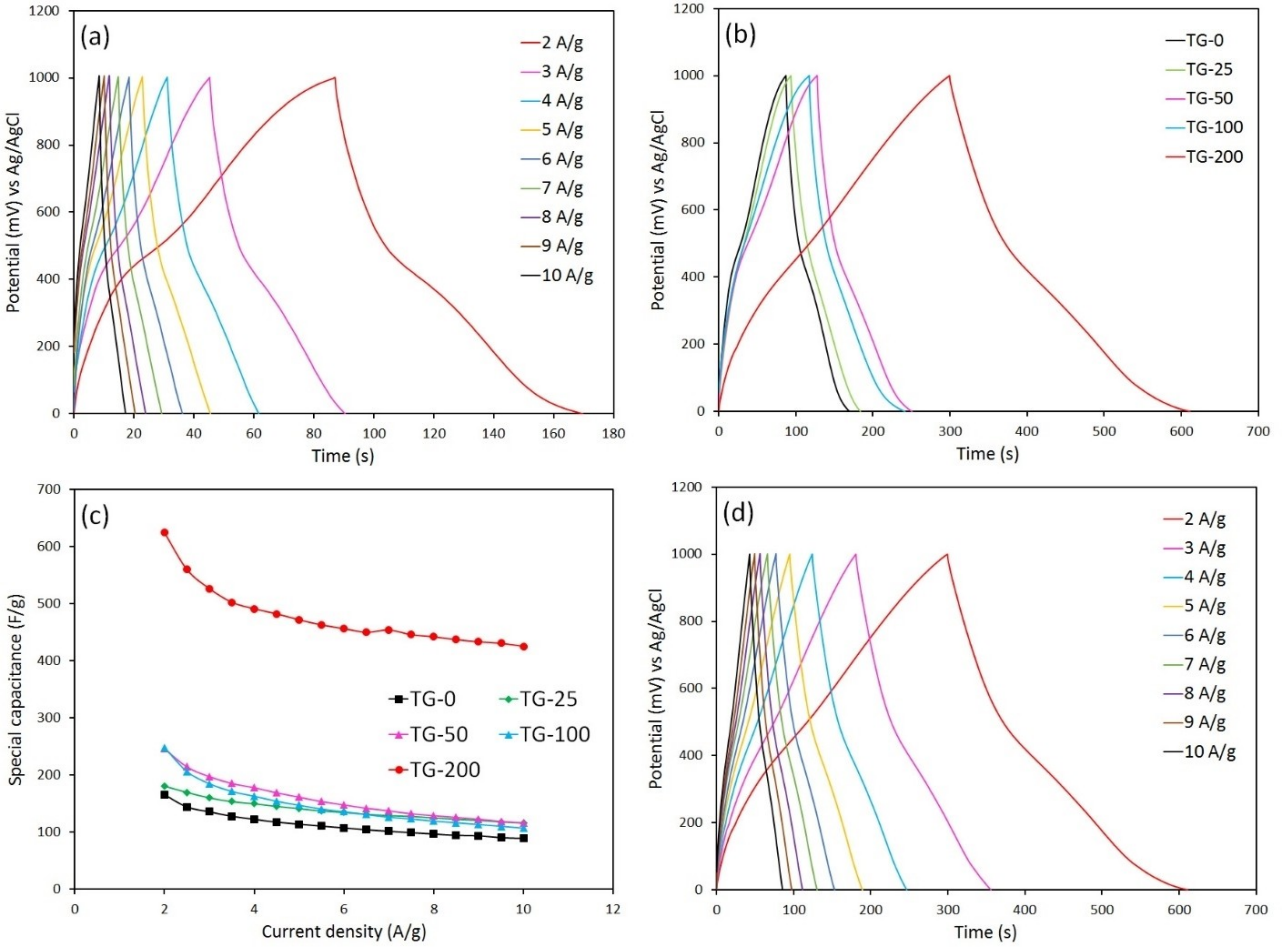
GCD curves of (a) TG‐0 at different current densities and (b) all nanocomposite samples at 2 A/g. (c) Variation of specific capacitance versus current density for different nanocomposite samples. (d) GCD curves of TG‐200 sample at different current densities.

The electrochemical performance of TiO_2_ and TiO_2_‐rGO samples was also studied by galvanostatic charge/discharge (GCD) analysis. The GCD curves of the TG‐0 sample in the potential range of 0–1 V at different current densities are shown in Figure 7(a). These curves exhibited nearly linear profiles and symmetric triangular shapes, suggesting favorable capacitive behavior, excellent electrochemical reversibility, and efficient charge/discharge kinetics. The specific capacitance of the electrodes can be determined from the GCD curves at different current densities using the following equation:[^21^, ^35^]
(1)

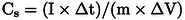




where I is the applied current, ▵t is the discharge time, m represents the mass of electroactive material used as the electrode, and ▵V is the voltage window. The specific capacitance values calculated for the nano‐TiO_2_ electrode range from *c.a*. 89 to 165 F/g at current densities of 10 and 2 A/g, respectively. The specific capacitance of TiO_2_ electrode at current densities of 2 A/g was previously reported to be 45 F/g^[21]^ and 135 F/g,^[25]^ indicating our TiO_2_ nanoparticles were synthesized with a high quality in terms of electrochemical behavior. Increasing current density leads to a decrease in charge/discharge time duration. At lower current densities, electrolyte ions can penetrate the inner surface of the electrode, thereby increasing the available surface area and enhancing the specific capacitance.

Figure 7(b) shows the GCD curves of different nanocomposite samples with varying graphene content at a current density of 2 A/g. The presence of graphene additive maintained the symmetrical triangular patterns in the GCD curves. Analysis of the GCD profiles revealed that the discharge time observed in TiO_2_‐rGO electrodes surpassed that of pure TiO_2_ electrode, consequently enhancing capacitive behavior and increasing specific capacitance as defined by Eq. 1. This improved electrochemical performance of nanocomposite electrodes is attributed to their enlarged surface area, serving rGO as an ion reservoir, providing more active sites, and facilitating faster electron transfer.^[9]^ Based on Figure 7(b), the TG‐200 sample exhibited the lengthiest discharge time among the samples studied. With a graphene content of 20 wt. %, this sample achieved the highest specific capacitance of *c.a*. 624 F/g at a current density of 2 A/g. Xiang *et al*.^[16]^ reported specific capacitance values of 62.8 and 225 F/g for their rGO‐TiO_2_ nanoparticles and rGO‐TiO_2_ nanobelts, respectively, with an rGO:TiO_2_ ratio of 7 : 3 at a current density of 0.125 A/g. In addition, the specific capacitance of TiO_2_ NWs‐rGO nanocomposite with a mass ratio of 1 : 4 reached about 550 F/g at a current density of 2 A/g.^[19]^ The specific capacitance values for TiO_2_‐rGO hybrids in other studies, where the mass ratios were unspecified, were around 380 F/g at a current density of 2 A/g^[21]^ and 500 F/g at a current density of 2 mA/g.^[25]^ Therefore, the C_s_ value obtained in our study stands out as one of the highest reported values to date, while the graphene content in this particular nanocomposite sample is among the lowest used in comparison to other works.

To evaluate the rate capability of nanocomposite samples, Figure 7(c) shows the variations of the C_s_ values of different nanocomposites with current density. As the current density increased, the specific capacitance of all samples declined, a phenomenon associated with the ion exchange mechanism. At lower current densities, electrolyte ions have enough time to fully interact with the active surface of the electrode material. Conversely, at higher current densities, electrolyte ions struggle to thoroughly penetrate into the electrode material due to insufficient charge transfer time. Notably, the TG‐200 sample exhibits the highest capacitance at all current density values. More graphene in the composition enhances the sample‘s conductivity, leading to a larger surface area and shorter ion travel pathways. The specific capacitance of this sample diminished from 624 F/g at 2 A/g to 425 F/g at a current density as high as 10 A/g, indicating its high rate capability. Figure 7(d) shows the GCD curves of the TG‐200 sample, identified as the specimen with the most exceptional capacitive performance, at different current densities.

**Figure 8:**
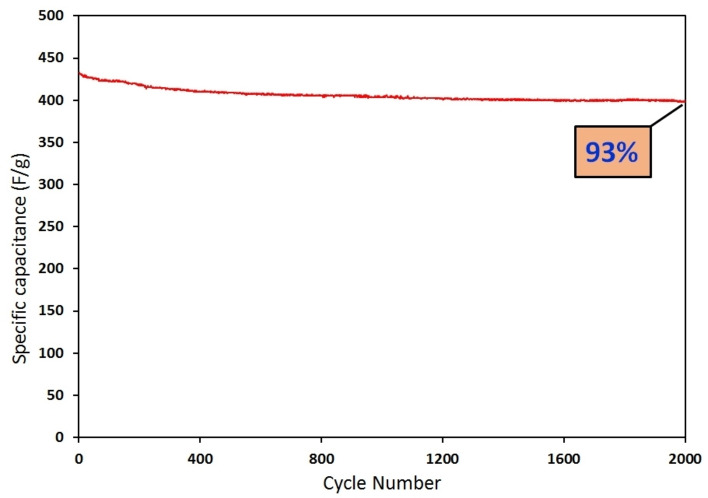
Cyclic stability of TG‐200 sample at 10 A/g in 2000 cycles.

Long cyclic stability is a crucial feature for supercapacitors that greatly influences their practical viability. The cyclic stability for the TG‐200 electrode was assessed by repeating the GCD measurements within a voltage range of 0–1 V at a constant current density of 10 A/g for 2000 runs, as shown in Figure 8. It was found that the nanocomposite electrode exhibited a specific capacitance retention of 93 % after 2000 cycles, demonstrating an excellent cycle life conductive for supercapacitor applications.

**Figure 9:**
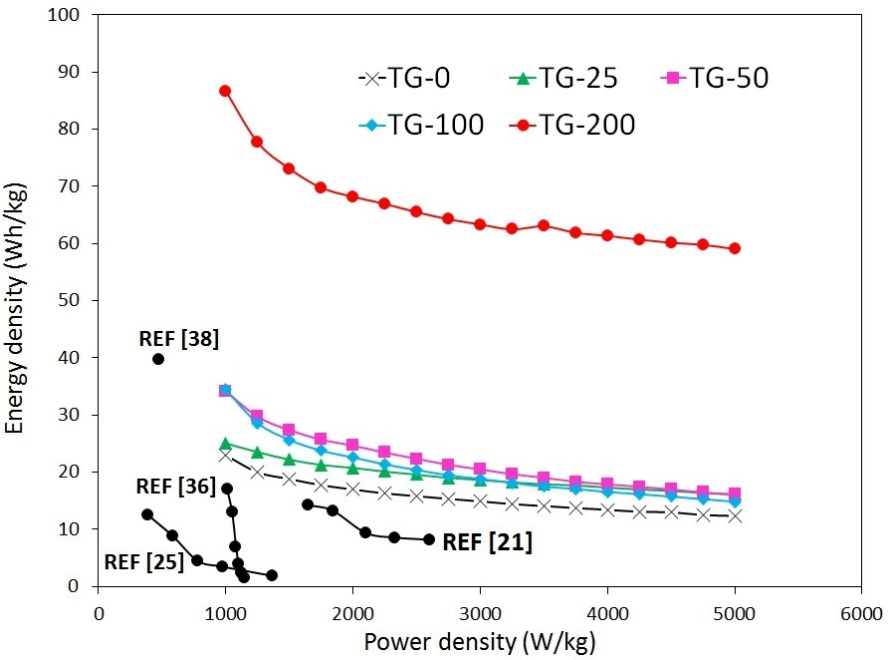
Ragone plot of the nanocomposites electrodes.

Electrochemical parameters including Coulombic efficiency, power density, and energy density are important to describe the overall performance of supercapacitors. The Coulombic efficiency can be determined by analyzing the GCD curves using the following equation:[^21^, ^35^]

(2) $$\eta =\;\left(\frac{t_{d}}{t_{c}}\right)\times 100$$

where *t_c_* and *t_d_* are charge and discharge time for a specific current density. The Coulombic efficiency values of all nanocomposite samples were in the range of 95 %, showing an acceptable symmetry between charge and discharge process. Also, the energy density (Wh/kg) and power density (W/kg) of the electrodes can be calculated using Eqs. 3 and 4:[^36^, ^37^]

(3) $$E=\;\frac{C_{s}{\Delta V}^{2}}{7.2}$$

(4) $$P=\;\frac{3600\times E}{t_{d}}$$

where *C_s_* is the specific capacitance (F/g), Δ*V* is the potential range (V), and *t_d_* is the discharge time (s). The Ragone plot in Figure 9 illustrates the energy density as a function of power density for pure TiO_2_ and nanocomposite electrodes. Based on the findings, the energy density values of all TiO_2_‐GO electrodes are higher than that of TiO_2_ electrode. At lower current densities, greater energy density is obtained due to the higher density of ions moving between electrolyte and electrode. On the other hand, at higher current densities, energy density decreases due to the increased resistance (R_ct_) and limited use of the electrode.^[25]^ The superior energy density in nanocomposite electrodes is also attributed to their greater specific capacitance values. The Ragone plot shows the power density ranging from 1000 to 5000 W/kg for current densities of 2 to 10 A/g, respectively. Furthermore, the TG‐200 sample has the highest energy density among the other nanocomposites, with values of 86.64 Wh/kg at 2 A/g and 59.03 Wh/kg at 10 A/g. In fact, increasing the graphene content resulted in an increase in the energy density due to its direct effect on the specific capacitance. It can be inferred that 2D materials like graphene, due to their sheet structure and the ease of transferring ions from the electrolyte to the electrode, can easily provide a higher specific capacitance and subsequently, a higher energy density. In addition, combination of graphene and oxide material, like TiO_2_, presents a promising option to achieve high‐performance electrochemical cells, primarily due to the mixed mechanism of EDLC and pseudocapacitance.[^39^, ^40^] The comparison of energy density and power density data between our study and existing literature (Figure 9) demonstrates the exceptional electrochemical performance of the cell developed in this study, positioning it as one of the most high‐performing supercapacitors using from this specific nanocomposite to date, to the best of our knowledge.

**Figure 10:**
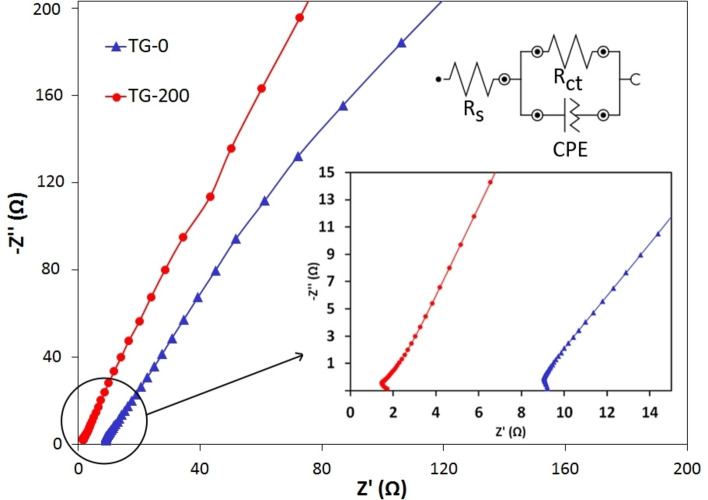
Nyquist plot of TG‐0 and TG‐200 samples.

The Nyquist plots of the pure TG‐0 and TG‐200 samples derived from EIS measurements are shown in Figure 10. The inset shows the curves in a magnified view near the origin. The impedance spectra exhibit a small semicircular arc in the high‐frequency domain and a straight line at low frequencies, confirming the hybrid characteristic of the supercapacitor, i.e. combination of pseudocapacitance and EDLC. The semicircles have an incomplete, depressed shape, and only a small portion of them are visible in the high‐frequency region. This indicates a non‐ideal capacitive behavior, which can be caused by factors like surface roughness, inhomogeneities, grains, grain boundaries, impurities or substrate/electrode interface.[^41^, ^42^] The straight line is identified as the Warburg resistance, associated with ion transfer/diffusion through the electrolyte to the electrode surfaces.[^25^, ^35^] This relatively vertical line in the low‐frequency region indicates the fast ion diffusion in the electrolyte and the adsorption of ions on the electrode surface. An inset in Figure 10 displays the equivalent electrical circuit, with R_s_, R_ct_ and CPE denoting the solution resistance, charge transfer resistance, and constant phase element, respectively. The R_s_ value for the TG‐200 sample (2.08 Ω) is lower than that of the TG‐0 sample (9.15 Ω), indicating enhanced accessibility of active sites on the nanocomposite surface for electrolyte ions.^[21]^ The R_ct_ values estimated from the semicircular arc for TG‐0 and TG‐200 samples were 1700 and 679 Ω, respectively. The TiO_2_‐rGO nanocomposite electrode exhibited lower R_ct_ compared to the pure TiO_2_, indicating the incorporation of TiO_2_ nanoparticles and the graphene nanosheets can result in an improved charge transfer phenomenon within the electrode. This improvement is mostly due to the higher surface area and more active sites provided in the nanocomposite sample as well as the high conductivity of graphene sheets, which can accelerate the charge‐transfer reactions.

## Conclusions

This study successfully synthesized TiO_2_‐graphene nanocomposite using hydrothermal method, which was confirmed by XRD, FTIR and Raman analyses. Surface morphology and microstructure investigation conducted by SEM and TEM revealed the uniform distribution of TiO_2_ nanoparticles within the graphene sheets. Various nanocomposite specimens with varying rGO content (2.5, 5, 10, 20 wt. %) were prepared, and the effects of graphene content on the supercapacitive behavior of the fabricated cells were investigated. It was found that the addition of graphene to TiO_2_ nanoparticles improved the electrochemical performance of the electrodes. Among nanocomposite samples, TiO_2_‐20 wt. % rGO exhibited the most favorable supercapacitive properties. Its specific capacitance values at current density of 2 and 10 A/g were 624 and 425 F/g, respectively, representing one of the highest capacitance and rate capability achieved by this composite. In addition, this nanocomposite sample showed a 93 % cyclic stability of the capacitance over 2000 cycles at 10 A/g. Using Ragone plot, the energy density and power density of the TG‐200 sample were determined to be 86.64 Wh/kg and 1000 W/kg, respectively, at a current density of 2 A/g.

## Experimental

**Chemicals**: Graphene oxide (GO) suspension and titanium tetraisopropoxide (TTIP) were purchased from Nano Mavad Gostaran Pars Co. (I. R. Iran). Polytetrafluoroethylene (PTFE) and carbon black powder were purchased from Merck Co. Ethanol was purchased from Dr. Mojallali Co (I. R. Iran). Deionized (DI) water was obtained by purification through a lab‐scale water purifier.

### Synthesis of TiO_2_ Nanoparticles

To synthesize TiO_2_ nanoparticles, initially, 1.6 ml of TTIP was added dropwise into 50 ml of DI water under vigorous stirring, leading to the turbidity of the solution caused by the formation of finely dispersed white particles. This suspension was heated at 150 °C in a 300 ml Teflon‐lined autoclave for 24 h. Afterwards, the resulting powder was dried in an oven at 80 °C. The dried powder was then subjected to calcination in a furnace at 550 °C for a duration of 90 min.

### Synthesis of TiO_2_‐rGO Nanocomposite

Graphene oxide was incorporated into TiO_2_ nanoparticles at varying weight fractions. The different samples were denoted as TG‐0 (pure TiO_2_), TG‐25 (TiO_2_‐2.5 wt. % rGO), TG‐50 (TiO_2_‐5 wt. % rGO), TG‐100 (TiO_2_‐10 wt. % rGO) and TG‐200 (TiO_2_‐20 wt. % rGO). Initially, appropriate amount of GO suspension, with a concentration of 4 g/l, was diluted with DI water and stirred for 15 min. Subsequently, TTIP was added dropwise and stirred for another 15 min. This suspension was transferred to a 300 ml Teflon‐lined autoclave and maintained at 150 °C for 24 h, resulting in the formation of a black gel. In the next step, the gel was dried at 80 °C for 90 min. Finally, calcination was performed at 550 °C for 90 min under a nitrogen atmosphere.

### Characterizations

The morphologies of TiO_2_ nanoparticles and TiO_2_‐rGO nanocomposite were examined using a field‐emission scanning electron microscope (FE‐SEM, MIRA3 TESCAN) and a transmission electron microscope (TEM, Philips EM 208 s) operating at a voltage of 100 kV. Fourier‐transform infrared spectroscopy (FTIR) analysis was conducted with a PERKIN Elmer 1720‐X model. The Raman spectrum (excited at 514 nm) was recorded at ambient temperature using a micro‐Raman system (TEKSAN Co.). X‐ray diffraction (XRD) analysis was carried out by a GNR Explorer X‐ray diffractometer using Cu Kα radiation, a tube voltage of 40 kV, and a tube current of 40 mA.

To evaluate the electrochemical performance of the synthesized nanocomposite, a 3‐electrode system was utilized, comprising an Ag/AgCl electrode as the reference electrode, a platinum rod as the counter electrode, the synthesized active materials as the working electrode and 1 M H_2_SO_4_ as the electrolyte solution was considered. For preparation of the working electrode, a mixture of synthesized materials, carbon black and PTFE with a fixed weight ratio of 80 : 15 : 5 was dispersed in NMP and sonicated for 20 min to achieve a uniform paste. This paste was then coated onto graphite substrates (25×15 mm^2^) and dried at 80 °C for 4 h. Subsequently, the fabricated electrodes were subjected to cyclic voltammetry (CV), galvanostatic charge‐discharge (GCD), and electrochemical impedance spectroscopy (EIS) tests. The CV measurements were conducted at various scan rates (5–500 mV/s) within a potential range of 0–1 V. The GCD measurements were also performed at different current densities (2–10 A/g) with a cut‐off potential set at 1 V (vs. Ag/AgCl reference electrode). The EIS measurements were executed over a frequency range of 0.01 Hz–100 KHz using the open circuit potential (OPC) technique with an AC perturbation of 10 mV.

## Conflict of Interests

The authors declare no conflict of interest.

1

## Data Availability

The data that support the findings of this study are available from the corresponding author upon reasonable request.
